# Guess Work in the Practice of Medicine1Read at the forty-first annual meeting of the Medical Association of Central New York, held at Buffalo. October 27, 1908.

**Published:** 1909-07

**Authors:** B. C. Loveland

**Affiliations:** Syracuse, N. Y.


### Guess Work in the Practice of Medicine[1]

BY B. C. LOVELAND M. D., Syracuse, N. Y.

IN selecting a subject for this paper my mind ran over the various cases which have recently been of unusual interest to me, but after considering the matter carefully, I became convinced that a paper with the above title would give me an opportunity to say something which might be of more value to the profession than any dissertation I might make on any special disease or mode of treatment.

A number of years ago I became familiar with a maxim which runs: "A thing that is worth doing is worth doing well."

Applying this principle to the practice of medicine, I was led to believe that when I was consulted by a patient for a complaint, no matter how apparently trivial the complaint might be, I was consulted with the hope of securing relief from the complaint, and that it was clearly my duty to give the matter sufficiently serious attention and investigation to secure the desired result when such was possible. The case might be as plain as a boil on the end of the nose, and might take but a moment to make a diagnosis and outline treatment, or it might be some obscure internal condition, the correct understanding of which would require much time, and thought, and possibly more than one interview.

The apparent gravity of the case should not alter the conception of duty, and in any case nothing which would add to positive knowledge of the condition should be overlooked or left

---

1. Read at the forty-first annual meeting of the Medical Association of Central New York, held at Buffalo, October 27, 1908.

undone, and nothing left to guess work which could possibly become positive knowledge. The physician, then, should not be governed by appearances alone, or because he is busy simply listen to a story of suffering, more or less clearly told and at once conclude to give the patient a tonic or a little pepsin, then show the patient out of the office with the remark: "I think this will help you out. If you are not all right in a day or two come in and see me again." This does not fulfil the physician's obligation to his patient. It is this kind of try-this-and-if-you-do-not-get-better-come-again prescribing that this paper is directed against.

But you may say such prescribing represents a practice most infrequent in the medical profession, and only then in things either absolutely plain, or insignificant in importance. I contend that the boil on the nose may not be so simple as it looks, for it has more than once led me to an investigation which disclosed diabetes, and no man can say that a thing he has only observed superficially is or is not insignificant.

I am ready to admit also that occasionally a patient is to blame for the superficial attention given by the doctor, as an instance I will relate shows: July 13, 1901, I was consulted by Mrs. N. She told me she was troubled with dyspepsia and wanted me to give her something for it. She was 60 years of age and looked poorly nourished, said her bowels were all right, but she had some trouble with gas in her stomach after eating. I told her I had little use for guess work, and that before prescribing for her it would be wise to make a physical examination. She objected, possibly fearing an extra charge, but said "I am not sick enough for that. Just give me something and if I do not get better I will come and see you again." I did as requested, gave her something that I thought would help her, and a week later she reported better and wanted some more of the same medicine. After this I heard nothing for about a month when I was called to her house to find her very ill with an exhausting diarrhea, nausea and vomiting, both limbs edematous, and confined to bed. She had been away at a sanitarium where she expected to remain through a month or so of the hot weather, but after a hot bath or two she was completely prostrated, and her bowels began to move six or eight times a day as when I saw her. After nine days of this she was brought home in arms. Now, there was very little opposition to a reasonable investigation and I found her heart showed a mitral regurgitation with lack of compensation. Her liver was acutely congested and slightly enlarged. Her edema was due to these conditions. She had no albuminuria. She was tympanitic and tender over the whole extent of the abdomen. I will omit details of treatment as that does not come within the province of this paper, simply saying that she made a slow and tedious recovery, though permanent up to the present; but a proper study of her case at the beginning would have shown the heart lesion, the liver condition already begun and would have probably brought out the fact that her intestinal function had not been properly performed for months or perhaps years, for later it developed that she had had periods of costiveness alternating with periods of diarrhea for years, and that was what she called "all right" when I first questioned her, and this was but a step to chronic diarrhea. Such a frame of mind on the part of a patient is not unique, and frequently any reasonable insistance on the part of the physician will not change it. It is wise, however, to demand an opportunity to find out what the matter is before prescribing.

There is another class of patients whose troubles do not presage imminent dissolution, but who suffer much of many physicians, like the woman in the Scripture, and who often drift into a deplorable state before anyone takes sufficient time or interest in them to make a careful study of their troubles and set them on the right path again. This is the class which particularly excites my interest, and which has prompted this paper. An example or two of this sort will be briefly reported here for purposes of illustration.

Mrs. F. B., widow of a physician who had practised in a neighboring town consulted me on September 5, 1902. She was 65 years of age, had suffered with eczema for 20 years, was very thin, poorly nourished, and of a peculiar purplish brown complexion. Her eyes were much congested, and there was a phlyctenular ulcer on the margin of one cornea. Her bowels were loose, each movement being preceded by pain in the region of the navel, and followed by a feeling of great exhaustion. At other times she felt comparatively well and strong, though she had for years shown a bad color and suffered much as described. After taking her history, I requested a physical examination, to which she readily consented, and found a general entroptosis, the lower border of the stomach being on a level with the umbilicus, the right kidney nearly down to the pelvic brim, a sensitive liver, but no organic trouble. At this time she told me that she had consulted a large number of physicians in a number of places, and no one had ever made a physical examination, the nearest to it being when a physician in New York asked her to lie on a sofa in his office and palpated over her abdomen, without asking her to loosen or remove any of her clothing. With this item of history in mind, it was not strange that she had "doctored twenty years with no material benefit."

Her improvement under appropriate treatment was fairly satisfactory, apparently quite satisfactory to her, for up to the present time she says she is "very well," though she still retains much the same bad complexion.

Mrs. C. E. B., 38 years of age, called on me July 23, 1908. She was the mother of two children, the youngest 15 years old, had one miscarriage ten years ago. Father alive at 72 years of age, mother died of tuberculosis at 41. Thirteen years ago she began to feel as if her throat and the passage up to her right ear was closing up, "and it made her very nervous." Her family physician not succeeding in giving her relief, she consulted a laryngologist who did some operation in her throat. She said "tore loose a spasmodically contracted muscle." No beneficial results following, she consulted another laryngologist who operated on both inferior turbinated bones, and inflated her Eustachian tube several times with no benefit. Then she went to another laryngologist who told her that no more operating would benefit her, and after a few visits said he could not do her any good, and under the circumstances did not want to run up a bill against her. Later, discovering a swelling in her breasts, became convinced she had cancer, and both breasts were amputated, successfully, no cancer returning. Her old trouble in the throat still continued, and she tried other specialists, oculists, osteopaths, and finally another surgeon who recognised that the case was out of his province and sent her to me. An examination disclosed no trouble in the throat, nose free on both sides, though she felt as if the right nostril was closed, no sign of return of growths in territory invaded by the operations on her breasts, a very poor nutritional state, normal reflexes, and station, though she complained of a feeling of tremor and dizziness. Pulse 100. Pressure with the finger on the right carotid gave relief to the choking sensation and also the feeling in the right ear and nostril, which remained while pressure was continued. She was constipated and suffered from cold extremities, so that she was sleeping under heavy blankets in the hottest summer weather, and her mental state was such that her troubles were constantly uppermost in her thought and conversation. When I tried to direct her attention to such physiologic helps as would build up her physique, and at the same time divert her attention from her throat I found that her throat was the center around which her whole life revolved, and she "would stand anything" if I could only do something which would stop that feeling in her throat.

Now this is the class of cases which furnish material for christian science, mind cures and Emanuel movement clinics and if their true character was recognised before too many physicians and surgeons had failed to bring relief, but succeeded in producing a general loss of confidence on the part of the patient in the practice of medicine in general, the proper psychotherapeutic measures might be applied without waiting for the various medical excrescences to bring relief, and much to the credit of our good reputation as well. It should not require a neurologist to recognise the hysteria in such a case, and it is gratifying that at least two specialists turned her away without further operating, though possibly the right carotid might have been tied another experiment in hope of relief. But I might say here that I do not approve of operative treatment of non-surgical disorders such as hysteria.

Since I began to write this paper, I heard a physician say in one of our hospitals that to make a successful practitioner, a man did not need much knowledge of anatomy, or pathology, or even to be a good diagnostician. All he needed was to know how to manage his patients. While I was willing to admit the value of being able to manage patients, and that a large number would recover anyway as he said, I had a very different idea of success than the remark quoted would imply, and asked him what of that relatively small number who will not "get well anyway?" He said "They will die anyway." But such is not the case, for many persons live to become chronic invalids for years, who with a correct diagnosis and proper management ought to be useful men and women.

I think this is sufficient in the way of illustrations to show the kind of practice of medicine this paper is directed against, that is, a practise marked by carelessness, indifference, inefficiency and guess work. That this kind of practise is all too common, and works discredit to our profession is a matter of daily observation, as much of my work is with patients who have been ailing and doctoring for months or years, often with many physicians. This discredit to the medical profession is not the result of the occasional disastrous result to the patient, for it is proverbial that when one is dead he is soon forgotten, but it often happens that a person lives and goes round saying "I doctored six months with Dr. So and So, one of your leading physicians, and was no better but rather worse at the end of that time," or as mentioned before, "I have been from doctor to doctor for five years, and one after the other has told me to try this or that, and none of them have really helped me, and no one has ever carefully examined me. I think they did not know anything about my trouble but were just experimenting."

These are the things which breed lack of confidence on the part of the public, prevent proper medical legislation, and foster the growth of every ism and pathy, and furnish a field for the quack, and advertising charlatan who announces "consultation free and a positive diagnosis of your trouble." I hope I shall not be misunderstood as arraigning the whole profession in this regard, for that is not my intention, and I believe that no more careful and conscientious body of men exists than our profession as a whole, yet the evil mentioned is sufficiently common to make a point for my paper, and it is in the interest of that large body of careful workers that this subject is discussed, for the general medical body suffers more or less, as does the human body, when any member suffers, or imperfectly performs its function.

402 EAST GENESEE STREET.

